# Neoadjuvant Gemcitabine Chemotherapy followed by Concurrent IMRT Simultaneous Boost Achieves High R0 Resection in Borderline Resectable Pancreatic Cancer Patients

**DOI:** 10.1371/journal.pone.0166606

**Published:** 2016-12-09

**Authors:** Xiaolun Huang, Jeanna L. Knoble, Ming Zeng, Fernando N. Aguila, Tara Patel, Lowell W. Chambers, Honglin Hu, Hao Liu

**Affiliations:** 1 Department of Hepatobiliary-Pancreatic Surgery and Cell Transplant Center, the Affiliated Hospital of University of Electronic Science and Technology, Chengdu, Sichuan, China; 2 Department of Hematology and Oncology, Zangmeister Cancer Center, Mount Carmel Health System, Columbus, Ohio, United States of America; 3 Department of Radiation Oncology, Mount Carmel Health System, Columbus, Ohio, United States of America; 4 Central Ohio Surgical Associates, Inc., Columbus, Ohio, United States of America; 5 Cancer Center, the Affiliated Hospital of University of Electronic Science and Technology, Chengdu, Sichuan, China; The University of Texas MD Anderson Cancer Center, UNITED STATES

## Abstract

**Background:**

To study the feasibility of down stage the borderline resectable pancreatic cancer (BRPC) to resectable disease, we reported our institutional results using an intensity-modulated radiation therapy (IMRT) simultaneous integrated boost (SIB) dose escalation approach to improve R0 resectability.

**Methods:**

We reviewed our past 7 years of experience of using neoadjuvant induction chemotherapy with Gemcitabine followed by concurrent chemoradiaiton for BRPC. During the concurrent, chemo was 5-FU and radiation were IMRT with SIB technique to target the key areas with dose escalation to 5600 in 28 fractions. The key areas were defined by PET positive area. This was followed by restaging imaging to rule out distant metastases before resection.

**Results:**

25 finished dose escalation protocol. 2 of the 25 cases developed distant metastases, 23 (92%) patients without distant metastases underwent pancreatectomy. Among the those received pancreatectomy, 22 (95%) achieved negative margin (R0). The gastrointestinal toxicity > grade 2 was 8% and there was no grade 4 toxicity.

**Conclusion:**

Neoadjuvant Gemcitabine-based induction chemotherapy followed by 5-FU-based IMRT-SIB is a feasible option in improving the likelihood of R0 resection rate in BRPC without compromising the organs at risk for toxicity.

## Introduction

For locally advanced non-metastatic pancreatic cancer, surgical resection offers the best cure rate. However, at diagnosis, up to 40% of cases are unresectable due to the tumor’s direct invasion into adjacent critical structures, particularly the major arteries, such as celiac and superior mesenteric vessels. Among patients with unresectable disease, there is a subgroup of patients with less local invasion with potential conversion from neoadjuvant treatment categorized as borderline resectable pancreatic cancer (BRPC) group. We used the following criteria to define BRCP patients: 1) focal tumor abutment of the superior mesenteric artery, 2) encasement of the gastroduodenal artery up to the hepatic artery, 3) or involvement of the superior mesenteric vein/portal vein that is potentially resectable and amenable to reconstruction.

Improving the outcome of pancreatic cancer and maximizing the convertibility from non-resectable borderline non-metastatic pancreatic cancer to resectable disease has become the recent focus of multidisciplinary tumor management. Various treatment approaches have been attempted in the past, such as: 1) using induction-dose intensity chemotherapy to achieve high resectable rates [1,2], 2) increasing the radiation dose during the chemoradiation part of neoadjuvant treatment after standard induction chemotherapy [3], and 3) using radiation dose escalation upfront during concurrent chemoradiation without induction chemotherapy [4]. The introduction of intensity-modulated radiation therapy (IMRT) with a simultaneous integrated boost (SIB) enables us to provide dose escalation to the gross tumor volume only during the 4D plan without substantially increasing the dose to the organs at risk or extending the radiation duration. PET scan as biological marker allows functional guiding to the target and are essential for biological based IGRT [4,5]. Although IMRT-SIB technique has been used at other sites of disease, very little has been shown about its usage to improve resectability of pancreatic cancer. We have been using this technique in an effort to achieve more durable local control for patients whose disease is either borderline resectable or medically inoperable non-metastatic locally advanced pancreatic cancer. The purpose of this study was to review our institutional experience with the IMRT- SIB to improve the R0 resection convertibility of locally advanced pancreatic cancer. We hypothesized that the higher dose area to tumor direct invasion of the major vessel would have tolerable toxicity and would provide much greater tumor shrinkage to enable complete R0 surgical resection.

## Patients and Methods

### Patient Characteristics and Neoadjuvant Treatment

Our Institutional Review Board approved this study. The patients included in this study were treated at our institution for locally advanced pancreatic cancer from February 2008 to May 2015. The inclusion criteria were pathologically confirmed ductal adenocarcinoma or intraepithelial carcinoma. 28 patients were eligible and enrolled in the IMRT-SIB treatment protocol. 25 patients completed the IMRT-SIB treatment and were included in this study.

### Protocol for Non-metastatic Pancreatic Cancer

**Fig 1:**
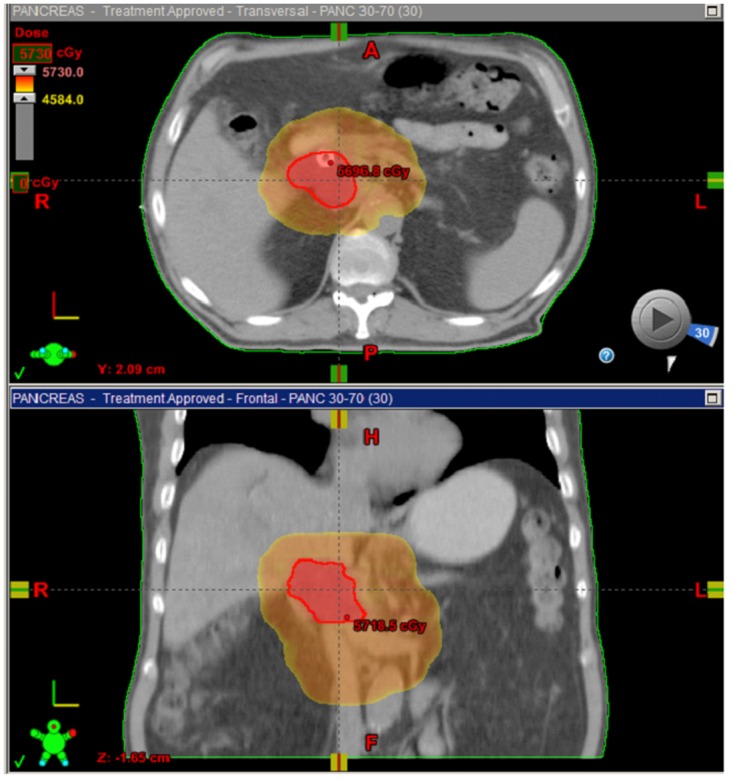
Axial and coronal images of representative IMRT-SIB. Treatment plan of using IMRT with SIB for pancreatic cancer summarized in the reprehensive images from treatment. Axial and coronal images of representative IMRT-SIB plans depicting dose wash of 5040 to CTV (pink) and dose escalation of 56 Gy to GTV (red).

**Fig 2:**
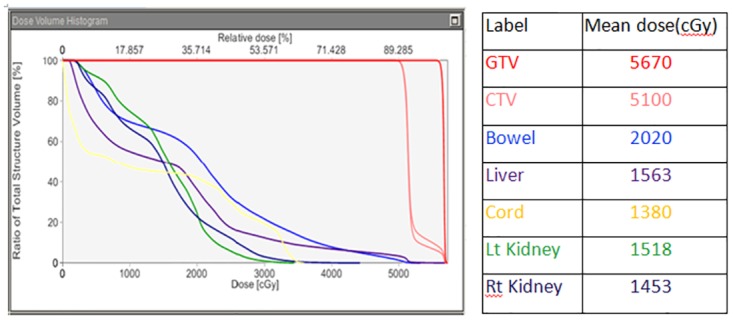
Dose volume histogram for the treatment plan with SIB. Dose volume histogram for the plan with SIB are showing here. The differential dose at the same large areas of CTV illustrated here. The higher dose in the area close to major vessel through SIB without increased the dose to the normal structures. The differential doses at the same areas of clinical tumor volume are illustrated. The mean doses (cGy) are: Gross Tumor Volume 5670; Clinical Tumor Volume 5100; Bowel 2020; Liver 1563; Cord 1380; Lt Kidney 1518; Rt Kidney 1453.

All treatment began with Gemcitabine-based cisplatin. Gemcitabine, IV: Initial: 1000 mg/m2 over 100 minutes on day 1 plus Cisplatin 25 i 40 mg/M^2^ over 120 minutes on day 2 for 3 weeks followed by 1 week rest; then once weekly for 3 weeks out of every 4 weeks for 2 cycles. After induction chemotherapy and chemoradiation, the patients were restaged. Computed tomographic and PET scans were performed approximately 1–3 weeks after completing induction chemotherapy. If there was no evidence of progressive disease, the patient received either CIV 5-FU (continuous infusion, 225 mg/M^2^) or oral capecitabine (capecitabine, Genentech, San Francisco, CA) 750 mg/M^2^ twice daily along with external beam radiation therapy (EBRT). The chemoradiation started within 4 weeks of completion of induction chemotherapy. The EBRT could be delivered through 4D vs IMRT with or without SIB. From 4–6 weeks post- chemoradiation therapy, patients underwent restaging workup for surgery. All patients who received radiation therapy started with computed tomography (CT)-based 4D treatment simulation. The simulation was performed with the patient in the supine position using immobilization with the patient’s arms over the head. The 4D simulations were performed if respiratory gating was feasible, otherwise, free-breathing 3D CT acquisition data would be obtained during simulation. All treatment planning in this series was performed by the same radiation oncologist. During the treatment planning, two target volumes were draw gross tumor volume and clinical target volume, PET/ CT obtained within 1–3 weeks from post-induction chemotherapy simulation data. Patients who did not have PET/CT data were excluded from the IMRT-SIB protocol. Clinical target volume, the clinical internal target volume, reflected the microscopic sites of highest risk. The treatment planning target volume for clinical target volume is about 5 mm beyond clinical target volume; the clinical target volume was contoured based on the Radiation Therapy Oncology Group consensus study protocol [6]. The treatment planning target volume for gross tumor volume will have no margins, see Figs 1 and 2.

### Surgical and Histopathologic Techniques

According to the neoadjuvant protocol, all patients received radiographic evaluation for distant metastases. Patients with non-metastatic disease were considered candidates for pancreatectomy using standard techniques. Surgical margins were designated in accordance with the criteria of the AJCC staging manual [7]. Margin status was confirmed by both frozen section and permanent section, the close distance to the nearest millimeter between cancer cells, and the margin was measured microscopically and recorded prospectively. The operation was defined as an R0 resection if there was no microscopic tumor found at the margin and as an R1 resection if a margin was microscopically positive. Segmental resection of the superior mesenteric artery, portal vein, or superior mesenteric vein/portal vein confluence was performed if the operating surgeon could not separate the pancreatic head from these vessels without leaving tumor on the vessel.

### Statistical Analysis

The data were analyzed using Pearson's Chi-Square tests to assess measures of association in the frequency table, P < 0.05 was considered to indicate statistical significance, and all statistical tests were based on a 2-sided significant level [8].

## Results

### Patient Characteristics

Patient characteristics are shown in Table 1. The mean follow-up period was 2 years 10 months. The median age at diagnosis was 65 years and 64% were men. The majority of patients began with an ECOG Performance Status 0 or 1 prior to starting therapy and 1 patient had a status of 2 due to brain injuries as child. Ninety-two percent of the tumors were adenocarcinoma.

**Table 1:** Characteristics of 25 Patients.

Characteristic	Value
Mean age (range)	65 years (45–82)
Men/women (n)	16/9
ECOG PS^a^ (n)	
0	16
1	8
2	1
3	0
Disease stage (n)	
I	0
II	3
III	22
IV	0
T stage (n)	
T1	3
T2	5
T3	17
N stage (n)	
N0	12
N1	13
Histology (n)	
Ductal adenocarcinoma	23
Intraepithelial carcinoma	2
Chemotherapy type (n) (Induction Gemcitabine 2 cycles)	
With cisplatin	17
With oxaliplatin	3
Induction GTX alone 3 cycles	2
Concurrent chemotherapy (n)	
5-FU	16
Capecitabine	9
Tumor location: head/body (n)	19/6
Mean gross tumor volume (cm^3^) (range)	43.7 (36.3–61.5)
Mean clinical tumor volume (cm^3^) (range)	513.2 (469.3–573.5)

^a^ Eastern Cooperative Oncology Group performance status.

The time required to finish radiation treatment ranged from 28–42 days. Clinical tumor volume represents the conventional dose coverage, 5040 in 28 fractions and PET positive alone the target area closing the major vessel will receive SIB to 5600 in 28 fractions, which labeled as gross tumor volume. Different areas received differential dose, with the high-risk areas receiving much higher dose per fraction, thus, the organs at risk were not compromised in terms of dose constraints. The average beam number was 5.5 (range, 5–9. The average time from last chemotherapy treatment and concurrent chemoradiation time was 31 days (range, 21–44 days). One patient received Gemcitabine during concurrent chemoradiation. All organs at risk met their dose constraints whether the IMRT with or without SIB.

Upon finishing the neoadjuvant protocol, all 23 patients were assessed through enhanced CT scan, 2 out of 23 (9%) were assessed through PET/CT. The criteria used for CT is to exclude patients who have developed progressive disease during the chemoradiation. Progressive disease was determined using standard Response Evaluation Criteria in Solid Tumors (RECIS)[9]. Table 2 shows the results of radiographic evaluation in patients with non-metastatic disease. Patients with stable and progressive disease who received the IMRT-SIB protocol treatment had significantly better response compared with patients not receiving the protocol treatment. Among the finished protocol patients, 6 out of 23 (26%) had a partial response, 15 out of 23 (65%) had stable disease, and 2 out of 23 (9%) had progressive disease (distant metastases disease to liver, 1 had R1 resection).

**Table 2:** Non-metastatic patient distribution per radiographic evaluation.

Response	SIB n (%)	Without SIB n (%)	X^2^	P value
Partial	6 (26)	5 (11)	0.182	0.5
Stable	15 (65)	31 (69)	11.13	0.001^a^
Progressive	2 (9)	9 (20)	9.909	0.004^a^
Totals	23 (100%)	45^b^ (100)	n/a	n/a

^a^ Statistically significant.

^b^ Eight additional patients had metastases to other sites and were not included in this analysis

In our cohort, 23 patients underwent surgery eventually. One patient with pancreatic pseudocyst developed local pain and radiographic enlargement and underwent surgery, which confirmed the pseudocyst rather than progressive disease. Since there is no clear evidence that a radiographic response to the neoadjuvant therapy for such a patient group is closely linked to the resectability, our results were consistent with previous reports [10].

### R0 Resection Evaluation

Of the 25 patients, 23 non-metastatic BRPC patients underwent Whipple resection with curative intent for primary adenocarcinoma of the pancreas. Most of the tumors were localized in the head of the pancreas and the most common histology was ductal adenocarcinoma. Pathologic assessment of 6 margins (proximal and distal superior mesenteric artery, proximal and distal superior mesenteric vein, pancreas, retroperitoneum, common bile duct, and hepatic artery) was undertaken by either frozen section (pancreas and common duct) or permanent section. A margin was considered positive if tumor was present < 1 mm from the inked specimen. Margins noted to be positive on frozen section were resected whenever possible. Of the 23 patients who underwent surgical resection, 22 (96%) had negative margins with R0 resection (X^2^ 0.008; P > 0.05). Patients who had positive margins (R1 resection) underwent postoperative chemotherapy (Table 3). Our R0 resection rate is consistent with the rates reported by Takahashi et al [11], and Mellon et al.[3](Table 4)

**Table 3:** Radiographic Evaluation for Response and Surgical R0 resection rate.

	Response patient (n) and rate	R0 Resection patient (n) and rate*
PR	6 (26.1)	6 (27.3)
SD	15 (65.2)	14(63.6)
PD	2(8.7)	2(9.1)
	23(100)	22(100)

PR: Partial Response, SD: Stable Disease, PD: Progressive Disease.

* χ^2^ = 0.008, *P*>0.05, there is no significant difference between the two groups.

**Table 4:** Published experience with neoadjuvant approach and R0 resection for localized pancreatic cancer.

Variable	Mehta 2001	Takahashi 2013	Esnaola 2014	Mellon 2015	Current report
Patients n	15	80^a^	37	110^b^	25
Induction Chemo	NA	Gem	NA	Fol	Gem
Radiation Dose/fx	5040~5600/28	5040/28	54/25	40/5	5600/28
Radiation Technique	NA	IMRT	IMRT	SBRT	IMRT-SIB
Radiation Sensitizer	FU	FU	FU	NA	FU
Resected n	9	NA	25	NA	23
R0 Resected n (%)	9 (60)	42 (98)	25 (69)	(96)	22 (96)

Abbreviations: Gem, Gemcitabine; FU, fluorouracil, NA, not applicable; FX, fraction; Fol: FOLFOX.

^a^ resectable and BRPC.

^b^ BRPC and locally advanced

### Toxicity Assessment

The toxicities related to neoadjuvant therapy did not prevent any of the patients from completing treatment or cause any subsequent surgical morbidity. Temporary toxicities related to treatment consisted of grade 0–1 colitis (n = 23), grade 2–3 colitis (n = 2), mild skin reaction/dermatitis (n = 3), abdominal pain and nausea (n = 8), febrile neutropenia (n = 14), and thrombocytopenia (n = 12). Our severe toxicity rate including grade 2 or 3 colitis was 8%, which is much lower than the reported rate of 34% for those using Gemcitabine[12] and comparable to other 5-FU regimens[13]. There was 1 postoperative complication (wound infection). There were no cases of liver or renal failure and there were no deaths related to neoadjuvant therapy or surgery. All patients who were admitted for surgery were discharged home.

## Discussion

Long-term survival after a diagnosis of pancreatic cancer is best achieved in patients who are diagnosed prior to the development of distant metastases and who are able to have R0 surgery. Multimodality neoadjuvant therapies have had the greatest impact on these patients by obtaining the maximal likelihood of tumor shrinkage resulting in R0 status after preoperative treatment[14]. Our protocol has combined Gemcitabine-based induction chemotherapy followed by IMRT-SIB with tumor mass only dose escalation. The induction Gemcitabine-based chemotherapy was used to screen those who had already developed distant metastases and to improve resectability.

There are several potential advantages of neoadjuvant radiation dose through SIB for non-metastatic BRPCs. First, the SIB dose only delivers to PET-positive areas with high potential for radiation resistance, which could result in more biological damage to tumor stem cell and inhibit the stem cells’ migration to other sites[15,16]. Second, SIB allows higher dose within the same treatment time as conventional radiation treatment, which has greater biological benefit by counteracting tumor repopulation and enhancing the biological effective dose [17, 18]. Third, the SIB allows lower doses to the organs at risk, such as kidney, large bowel, duodenum, stomach, and intestines, which has less dose change to surrounding tissue without increasing surgical wound healing process, making the protocol much easier to tolerate. Fourth, and more importantly, the higher dose to the higher risk area defined by PET could shrink the tumor and convert more from BRPC into R0 resectable status. Our results suggest the IMRT-SIB improved the resectability compared with conventional EBRT in our cohort.

The correlation between radiographic response assessment and pathological response has been well reported.[9] Table 3 compares published studies with our study. In our study, 93% of the patients finished the neoadjuvant protocol with either partial response or stable disease, which is consistent with other reports[9]. Our disease progression rate was 9%, superior to the 21% rate reported by in Assifi et al.[19], although this could be due to patient selection difference. The decision regarding which modality (chemotherapy vs radiation) is more controversial in the neoadjuvant setting. Both chemotherapy and radiation could reduce the viable tumor and improve local control. Our finding suggested that 10% higher radiation dose to PET-positive tumor mass compared with the report by Murphy et al[12]. resulted in a 95.6% resectable rate. Whether the dose escalation through IMRT- SIB alone achieved the high R0 resection rate remains to be confirmed by future study.

The resectable rate in our study is higher than reported by Esnaola et al[20]. which could be due to the difference of induction chemotherapy between the present study and Esnaola et al[20]. There is no consensus regarding which induction chemotherapy to use in this neoadjuvant setting. We used Gemcitabine-based induction chemotherapy, which may have contributed to the high resection rate. A recent report using FOLFIRINOX-based chemotherapy also achieved a high resection rate [3]. Which is better induction chemotherapy to convert resection rate for BRPC remains to be studied. The resectable rate in our study is comparable with the rate reported by Takahashi et al [11]. Our patients’ disease was more locally advanced as compared with the Takahashi cohort, which were resectable and borderline resectable patients. Interestingly, there is also no consensus regarding how to deliver radiation dose escalation in the neoadjuvant setting. Stereotactic Body Radiation Therapy has been utilized and Mellon et al[3].reported a high resection rate using this technique. It would be interesting to compare various techniques such as Stereotactic Body Radiation Therapy vs IMRT-SIB in neoadjuvant setting in a future study. To our knowledge, ours is the first report of using IMRT-SIB with dose escalation resulting in high convertibility. However, our study has limitations. First, this was a retrospective review; retrospective analysis mostly consisted of the patients who were treated by a dedicated multidisciplinary team at a single health institution. Selection biases from the study existed. Second, the sample size was too small to perform match pair analysis, which would provide much better assessment statistically to what extent that the SIB with dose escalation is responsible for improving the R0 resection rate. Moreover, although we have reviewed all pancreatic cases in our institute from the past 7 years, the IMRT-SIB has been implemented recently and, as a result, the follow-up for this subgroup of patients was short. In a prospective setting, the patients could be stratified by different prognostic clinical variables in an effort to better elucidate the role of SIB dose escalation in certain patient groups. For example, upcoming trial data from Radiation Oncology Therapy Group 1201 will provide more data regarding the role of dose escalation and its contribution to improving resection rate for these subset group patients. Last, R0 resection could be an important prognostic factor for better overall survival (OS), our cohorts remain in follow up, and the time is too short to complete the survival analysis at this juncture. The significance of using this strategy to achieve better survival for BRPC remains unclear.

## Conclusion

In summary, we report our Gemcitabine-based induction chemotherapy followed by IMRT-SIB-based dose escalation along with 5-FU-based concurrent chemoradiation for nonmetastatic border resectable pancreatic cancer. Our results suggest that this approach is an effective neoadjuvant regimen to achieve high R0 resection likelihood. Our analysis suggests that radiation differential dose escalation to the tumor mass, rather than microscopic disease, correlates with high resection rate. The approach has no higher toxicities and is well tolerated with acceptable administration and scheduling.

## References

[1] Krishnan S, Rana V, Janjan NA, Varadhachary GR, Abbruzzese JL, Das P, et al Induction chemotherapy selects patients with locally advanced, unresectable pancreatic cancer for optimal benefit from consolidative chemoradiation therapy. Cancer. 2007;110:47–55. 10.1002/cncr.22735 17538975

[2] Patel M, Hoffe S, Malafa M, Hodul P, Klapman J, Centeno B, et al Neoadjuvant GTX chemotherapy and IMRT-based chemoradiation for borderline resectable pancreatic cancer. J Surg Oncol. 2011;104:155–61. 10.1002/jso.21954 21520097 PMC3347705

[3] Mellon EA, Hoffe SE, Springett GM, Frakes JM, Strom TJ, Hodul PJ, et al Long-term outcomes of induction chemotherapy and neoadjuvant stereotactic body radiotherapy for borderline resectable and locally advanced pancreatic adenocarcinoma. Acta Oncol. 2015;54:979–85. 10.3109/0284186X.2015.1004367 25734581

[4] Ben-Josef E, Schipper M, Francis IR, Hadley S, Ten-Haken R, Lawrence T, et al A phase I/II trial of intensity modulated radiation (IMRT) dose escalation with concurrent fixed-dose rate gemcitabine (FDR-G) in patients with unresectable pancreatic cancer. Int J Radiat Oncol Biol Phys. 2012;84:1166–71. 10.1016/j.ijrobp.2012.02.051 22543215 PMC3421048

[5] Dirkx ML, van Sörnsen De Koste JR, Senan S. A treatment planning study evaluating a 'simultaneous integrated boost' technique for accelerated radiotherapy of stage III non-small cell lung cancer. Lung Cancer. 2004;45:57–65. 10.1016/j.lungcan.2004.01.003 15196735

[6] Jabbour SK, Hashem SA, Bosch W, Kim TK, Finkelstein SE, Anderson BM, et al Upper abdominal normal organ contouring guidelines and atlas: a Radiation Therapy Oncology Group consensus. Pract Radiat Oncol. 2014;4:82–9. 10.1016/j.prro.2013.06.004 24890348 PMC4285338

[7] Exocrine and Endocrine Pancreas In: Edge S, Byrd DR, Compton CC, Fritz AG, Greene FL, Trotti A, eds. AJCC Cancer Staging Manual, 7^th^ ed New York: Springer, 2010:241–246.

[8] Yates F. Contingency table involving small numbers and the X^2^ test. J Royal Stat Soc. 1934;1:217–235.

[9] Katz MH, Fleming JB, Bhosale P, Varadhachary G, Lee JE, Wolff R, et al Response of borderline resectable pancreatic cancer to neoadjuvant therapy is not reflected by radiographic indicators. Cancer. 2012;118:5749–56. 10.1002/cncr.27636 22605518

[10] Katz MH, Pisters PW, Lee JE, Fleming JB. Borderline resectable pancreatic cancer: what have we learned and where do we go from here? Ann Surg Oncol. 2011;18(3):608–10. 10.1245/s10434-010-1460-y 21136179

[11] Takahashi H, Ohigashi H, Gotoh K, Marubashi S, Yamada T, Murata M, et al Preoperative gemcitabine-based chemoradiation therapy for resectable and borderline resectable pancreatic cancer. Ann Surg. 2013;258:1040–50. 10.1097/SLA.0b013e31829b3ce4 23799421

[12] Murphy JD, Adusumilli S, Griffith KA, Ray ME, Zalupski MM, Lawrence TS, et al Full-dose gemcitabine and concurrent radiotherapy for unresectable pancreatic cancer. Int J Radiat Oncol Biol Phys. 2007;68:801–8. 10.1016/j.ijrobp.2006.12.053 17379445

[13] Kim EJ, Ben-Josef E, Herman JM, Bekaii-Saab T, Dawson LA, Griffith KA, et al A multi-institutional phase 2 study of neoadjuvant gemcitabine and oxaliplatin with radiation therapy in patients with pancreatic cancer. Cancer. 2013;119:2692–700. 10.1002/cncr.28117 23720019 PMC4174603

[14] Gillen S, Schuster T, Meyer Zum Büschenfelde C, Friess H, Kleeff J. Preoperative/neoadjuvant therapy in pancreatic cancer: a systematic review and meta-analysis of response and resection percentages. PLoS Med. 2010;7:e1000267 10.1371/journal.pmed.1000267 20422030 PMC2857873

[15] Kim CF, Jackson EL, Woolfenden AE, Lawrence S, Babar I, Vogel S, Crowley D, et al Identification of bronchioalveolar stem cells in normal lung and lung cancer. Cell. 2005;212:823–25. 10.1016/j.cell.2005.03.032 15960971

[16] Clarke MF, Dick JE, Dirks PB, Eaves CJ, Jamieson CH, Jones DL, et al Cancer stem cells—perspectives on current status and future directions: AACR Workshop on cancer stem cells. Cancer Res. 2006;66:9339–44. 10.1158/0008-5472.CAN-06-3126 16990346

[17] Cox JD, Pajak TF, Asbell S, Russell AH, Pederson J, Byhardt RW, et al Interruptions of high-dose radiation therapy decrease long-term survival of favorable patients with unresectable non-small cell carcinoma of the lung: analysis of 1244 cases from 3 Radiation Therapy Oncology Group (RTOG) trials. Int J Radiat Oncol Biol Phys. 1993;27:493–8. 8226140 10.1016/0360-3016(93)90371-2

[18] Machtay M, Hsu C, Komaki R, Sause WT, Swann RS, Langer CJ, et al Effect of overall treatment time on outcomes after concurrent chemoradiation for locally advanced non-small-cell lung carcinoma: analysis of the Radiation Therapy Oncology Group (RTOG) experience. Int J Radiat Oncol Biol Phys. 2005;63:667–71. 10.1016/j.ijrobp.2005.03.037 15927409

[19] Assifi MM, Lu X, Eibl G, Reber HA, Li G, Hines OJ. Neoadjuvant therapy in pancreatic adenocarcinoma: a meta-analysis of phase II trials.Surgery. 2011;150:466–73. 10.1016/j.surg.2011.07.006 21878232 PMC3164966

[20] Esnaola NF, Chaudhary UB, O'Brien P, Garrett-Mayer E, Camp ER, Thomas MB, et al Phase 2 trial of induction gemcitabine, oxaliplatin, and cetuximab followed by selective capecitabine-based chemoradiation in patients with borderline resectable or unresectable locally advanced pancreatic cancer. Int J Radiat Oncol Biol Phys. 2014;88:837–44. 10.1016/j.ijrobp.2013.12.030 24606850 PMC4120643

